# The Seed Region of a Small RNA Drives the Controlled Destruction of the Target mRNA by the Endoribonuclease RNase E

**DOI:** 10.1016/j.molcel.2012.07.015

**Published:** 2012-09-28

**Authors:** Katarzyna J. Bandyra, Nelly Said, Verena Pfeiffer, Maria W. Górna, Jörg Vogel, Ben F. Luisi

**Affiliations:** 1 Department of Biochemistry, University of Cambridge, Tennis Court Road, Cambridge CB2 1GA, England, UK; 2 Institute for Molecular Infection Biology, University of Würzburg, D-97080 Würzburg, Germany; 3 RNA Biology Group, Max Planck Institute for Infection Biology, D-10115 Berlin, Germany

## Abstract

Numerous small non-coding RNAs (sRNAs) in bacteria modulate rates of translation initiation and degradation of target mRNAs, which they recognize through base-pairing facilitated by the RNA chaperone Hfq. Recent evidence indicates that the ternary complex of Hfq, sRNA and mRNA guides endoribonuclease RNase E to initiate turnover of both the RNAs. We show that a sRNA not only guides RNase E to a defined site in a target RNA, but also allosterically activates the enzyme by presenting a monophosphate group at the 5′-end of the cognate-pairing “seed.” Moreover, in the absence of the target the 5′-monophosphate makes the sRNA seed region vulnerable to an attack by RNase E against which Hfq confers no protection. These results suggest that the chemical signature and pairing status of the sRNA seed region may help to both ‘proofread’ recognition and activate mRNA cleavage, as part of a dynamic process involving cooperation of RNA, Hfq and RNase E.

## Highlights

► Small RNA-mRNA duplex can recruit single-strand specific endoribonuclease RNase E ► sRNA can guide and allosterically activate RNase E to initiate target mRNA degradation ► The allosteric signal is a monophosphate group on the 5' end of the sRNA ► The 5' monophosphate may contribute to proofreading of sRNA action

## Introduction

In all bacterial species examined thus far, small noncoding RNAs (sRNAs) have been identified that act as post-transcriptional regulators of *trans*-encoded target mRNAs (Papenfort and Vogel, 2010; Gottesman and Storz, 2011). The sRNAs typically range in size from 50 to 300 nucleotides and encompass a short segment of 7 to 12 bases that pairs with a cognate region of partial or complete complementarity in the target mRNA. We will refer to this segment of contiguous base-pairing as the ‘seed region’ in loose analogy with the cognate region of eukaryotic microRNAs (Bartel, 2009). Despite the limited size of the pairing region, the sRNAs recognize targets with selectivity and affect rapid responses, which is most often by repressing translation and triggering degradation of the targeted mRNA. Many of the sRNAs studied in the model bacteria *Escherichia coli* and *Salmonella* sp. act in conjunction with the RNA chaperone Hfq, which assists the sRNAs to pair with their target mRNAs and also protects them from premature degradation (Vogel and Luisi, 2011). The main body of the sRNA might interact with Hfq such that the ‘seed region’ is presented to the transcript for cognate base-pairing (Papenfort et al., 2010; Balbontín et al., 2010; Sauer et al., 2012).

The primary nuclease through which sRNAs trigger transcript instability is the endoribonuclease RNase E (Caron et al., 2010; Aiba, 2007). While RNase E has little apparent sequence specificity, it nonetheless has strong preference to cut within single-stranded regions enriched in A/U (Carpousis, 2007). In *E. coli* and other alpha-proteobacteria, RNase E forms the scaffolding core of a multi-enzyme RNA degradative machine, known as the RNA degradosome, in which the enzymatic components can cooperate to turn over RNA (Górna et al., 2012). Once RNase E initiates cleavage, degradation proceeds rapidly for both mRNA and sRNA, so that their degradation is effectively coupled (Massé et al., 2003). The molecular events underlying the programmed degradation of the target, starting from the facilitation of the sRNA interaction by Hfq and concluding with the cleavage cascade by RNase E and the degradosome, are not well understood.

Two principle pathways may be envisaged to account for sRNA-induced mRNA decay. First, since many sRNAs act to repress translation, they disrupt the orchestrated synchrony of transcription and translation that shields and so stabilizes bacterial mRNAs. It is conceivable that sRNAs could destabilize target transcripts indirectly by simply depriving them of ribosomes, with the consequence that the exposed mRNA is freely attacked by RNase E (Wagner, 2009). The formation of the ternary complex of Hfq, RNase E and sRNA (Morita et al., 2005; Ikeda et al., 2011) could increase the local concentration of RNase E near the target mRNA to favor such attack. The recruited RNase E – in a constitutively active state – would have a greater opportunity to act on preferred cleavage sites either proximal (Afonyushkin et al., 2005) or distal (Prévost et al., 2011) to the site of sRNA pairing.

Another possible pathway of targeted gene silencing envisages that the sRNA/mRNA cognate pair actively stimulates RNase E to attack, as distinct from the simple, passive co-recruitment of the enzyme in a constitutively active state. According to this model, specific signals would trigger the ribonuclease to cleave an mRNA once it has been tagged by pairing of a cognate sRNA. Indirect evidence supporting this proposal comes from work on the sRNA MicC that was first identified as a repressor for expression of the *E. coli* outer membrane protein, OmpC (Chen et al., 2004). Recent analysis in *Salmonella* has shown that MicC also pairs within and induces cleavage of the coding sequence (CDS) of the transcript encoding the outer membrane protein OmpD, in conjunction with Hfq and RNase E (Pfeiffer et al., 2009). MicC binds the *ompD* mRNA far downstream of the RBS (ribosome binding site), at codons 23-26 where the short pairing of the sRNA is unlikely to abrogate translation (Bouvier et al., 2008). Consistent with its binding to a site remote from the RBS, MicC was found to be unable to inhibit translational initiation or elongation in vitro. In vivo, MicC expression induces a specific cleavage at position A+83 in the CDS of *ompD.* This product, which we are proposing results from activation of RNase E by sRNA, accumulates in the presence of C-terminal truncated RNase E that cannot assemble into the RNA degradosome (Pfeiffer et al., 2009).

The recruitment of RNase E in an activated state on a target mRNA could result, for instance, through the known ability of the ribonuclease for allosteric activation (Mackie, 1998). Data from X-ray crystallography, equilibrium binding and kinetics experiments show that RNase E family members recognize the 5′ end of certain substrates, and that this interaction boosts substrate affinity and triggers a structural change that organizes the active site and stimulates cleavage rates (Callaghan et al., 2005; Koslover et al., 2008; Jourdan et al., 2010; Jourdan and McDowall, 2008). RNase E possesses a 5′ sensing pocket (Callaghan et al., 2005; Garrey et al., 2009), and interaction of the 5′ end of the sRNA with this pocket could favor catalytic domain closure to boost the enzyme’s activity. We explored this hypothesis and present evidence in support of such a mechanism. We also present evidence that the 5′-end favors cleavage of the sRNA when it is not paired to the cognate transcript, and we discuss the mechanistic implications of this finding for the specificity of transcript targeting through a kinetic proofreading scheme.

## Results

### RNase E Activity Can Be Directed to a Defined Site on an RNA by the 5′ Monophosphate of an Artificial Guide RNA

**Figure 1:**
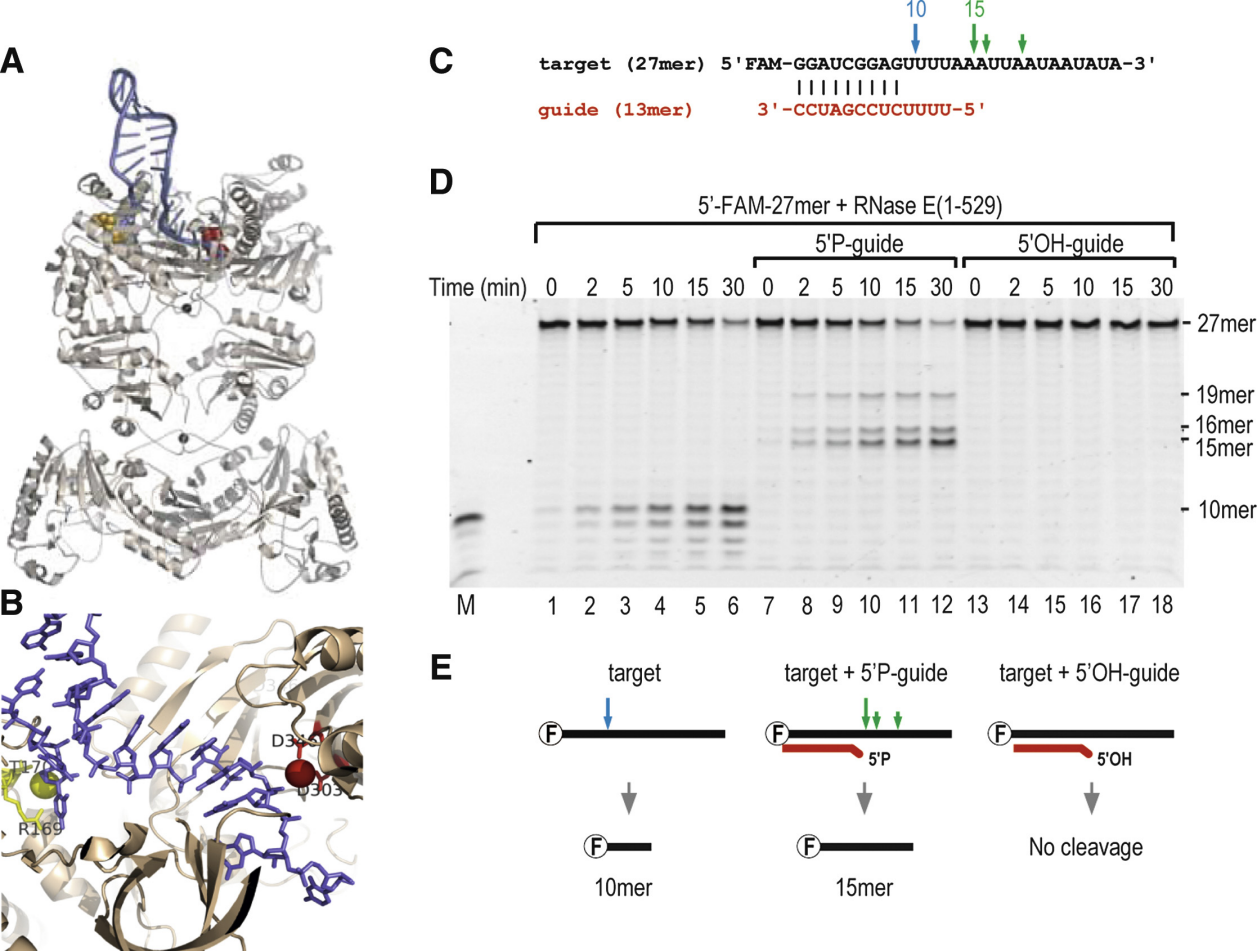
RNase E Cleavage Can Be Guided by an Artificial sRNA (A) Hypothetical model of the complex of the tetrameric catalytic domain of RNase E with a structured substrate, constructed from experimental crystal lattice interactions (Callaghan et al., 2005). (B) Expanded view that includes the 5′ sensor region and the catalytic site. The spacing from the end of the seed matching duplex region to the catalytic metal is 5 to 6 bases. The RNA substrate is colored blue; in yellow are the critical residues of the 5′ sensing pocket and 5′ phosphate atom, shown as a sphere; and in red are the active site aspartate residues and the catalytic magnesium ion (sphere). (C) The sequences of the 27-mer target RNA with a 5′ fluorescent group (FAM, black) and its partially complementary 13-mer guide (red). The guide has either a 5′ hydroxyl or a monophosphate group. (D) Denaturing gel showing the degradation products of 27-mer alone (left panel), in presence of 5′-P-13-mer guide RNA (middle panel) and in presence of 5′-OH-13-mer guide RNA (right panel). The visualization is for the FAM group so only the 5′end degradation products are visible. (E) Schematic of the cleavage patterns observed in (D). These are also indicated by arrows in (C) for the target 27-mer RNA in the presence (green) and absence (blue) of 13-mer guide RNA. (See also Figure S1.)

The crystal structure of RNase E catalytic domain in complex with 15-mer RNA (Callaghan et al., 2005) suggested a model for how the enzyme might cleave a structured substrate in which the 5′ phosphate is separated from a cleavage site by a secondary RNA structure (Figures 1A and 1B). In this way the RNA substrate (blue) would supply a single stranded 5′ end that could present a monophosphate group in the 5′ sensing pocket (yellow) to favor conformational adjustments that accommodate the 3′ single-stranded portion of the RNA in the active site (red). This model applies also to the duplex structure between two independent RNAs: the 5′ end of one RNA would act in *trans* to stimulate RNase E to cleave a second, accommodated RNA that is tethered through base-pairing. Such a situation is hypothesized to occur during sRNA-mediated cleavage of mRNA by RNase E.

To test the general principle, we used an artificial system comprising a target and a complementary guide (Figure 1C). In these experiments, a 27-mer RNA substrate was used that has an A/U rich segment which RNase E prefers to cleave (McDowall et al., 1994). The 27-mer is predicted to be predominantly free of secondary structure. The 5′ end of this RNA was labeled with a fluorescein group (FAM), and consequently the 5′ digestion products could be visualized by fluorescence imaging. When an excess of 27-mer was incubated with purified, recombinant RNase E catalytic domain (hereafter RNase E (1-529)), digestion products of 10 nts or shorter accumulate over time (Figure 1D, lanes 1-6). The cleavage occurs within the A/U rich region, in accord with the expected preferences of RNase E. The target 27-mer is protected from this attack by a 13-mer RNA with a 5′-OH group and 9 base pair complementarity upstream of the preferred cleavage site (Figure 1D, lanes 13-18). However, when the same 13-mer RNA has a 5′-monophosphate group, RNase E cleaves the 27-mer target at a new site that is six bases in the 3′ direction from the region of complementarity (Figure 1D, lanes 7 to 12; summarized in Figure 1E). This 6-base offset is consistent with expectations from the model based on the crystal structure (Figures 1A and 1B).

The cleavage patterns were not seen in controls using RNase E (1-529) inactivated by mutations in the catalytic site (supplementary materials, Figure S1) (Callaghan et al., 2005). If a small excess of the 27-mer over either 5′-P or 5′-OH 13-mer is used, the 10 nts product of the 27-mer alone becomes visible on the gel, suggesting that the 13-mer is affecting RNase E activity as a complex with the 27-mer and not on its own (result not shown). The reaction conditions have excess substrate over enzyme, so that the products are from multiple turnover events. The activating effect of a 5′-monophosphate was also seen when the 13-mer RNA has a 2′O-methyl modification throughout to protect against RNase E attack, showing that the directed internal cleavage of the 27-mer does not require cleavage within the 13-mer guide (Figure S1). The same guiding and activating effects were seen with purified recombinant RNase E catalytic domain from highly divergent bacterial species (*Caulobacter crescentus* and *Mycobacterium tuberculosis*; Steven Hardwick and Vivian Chan, unpublished results; data originated in Luisi lab), suggesting that 5′ end sensing by RNase E is conserved.

### The 5′ End of MicC Can Guide RNase E for Preferred-Site Cleavage of *ompD* In Vitro

**Figure 2:**
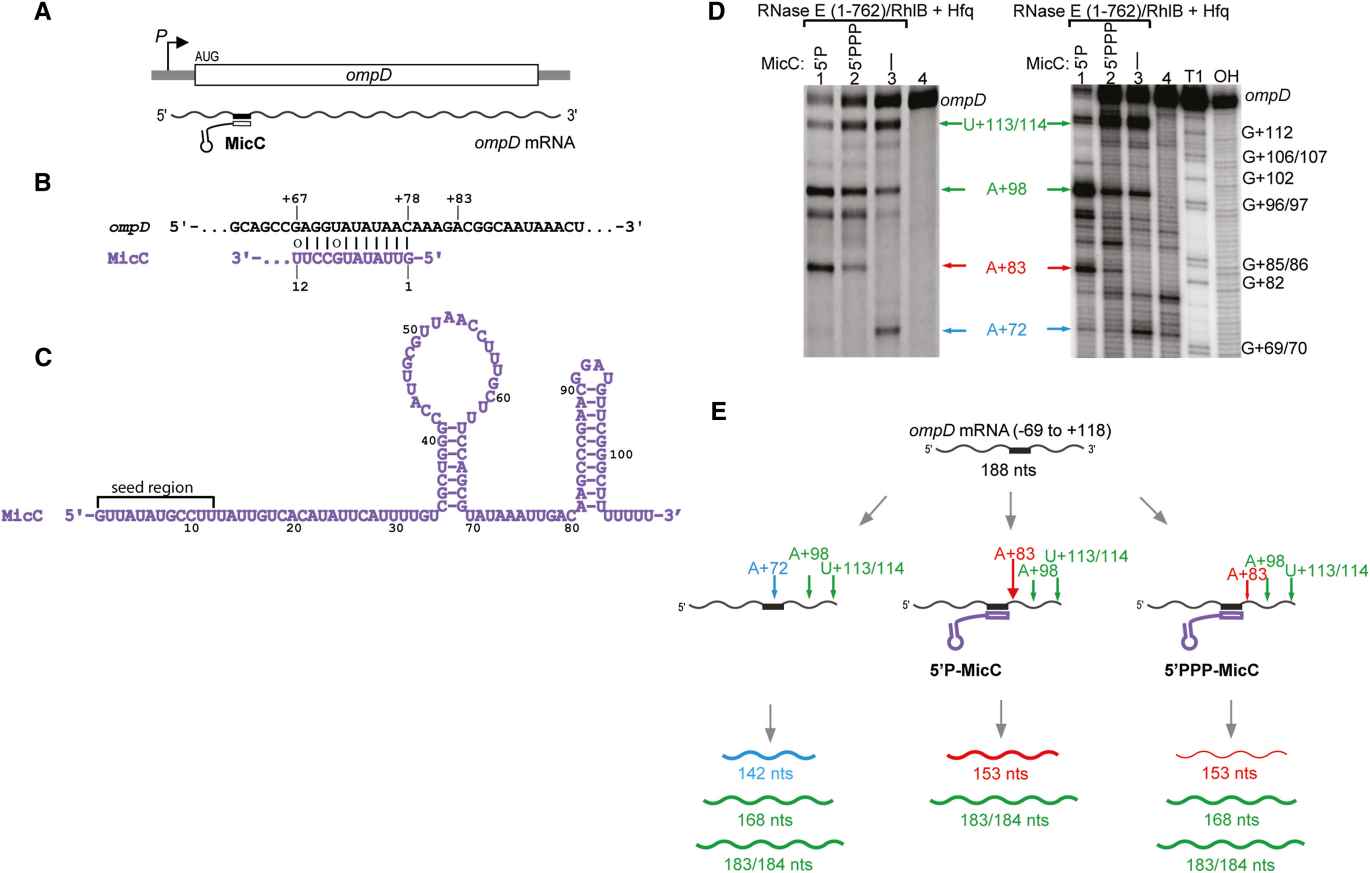
MicC Guides RNase E to Cleave *ompD* mRNA in the Coding Region (A) Schematic showing the site targeted by MicC within the *ompD* coding region. (B) The imperfect duplex formed by the 5′ seed region of MicC (purple) and *ompD* (black) showing the position of +83 nucleotide. (C) MicC secondary structure (Pfeiffer et al., 2009). The ‘seed’ recognition region is indicated. The polyU tail of sRNAs are important for mediating interactions with Hfq (Otaka et al., 2011; Sauer and Weichenrieder, 2011; Sauer et al., 2012). (D) (left panel) A denaturing gel showing main degradation products of *ompD* in vitro by RNase E (1-762)/RhlB and in the presence of MicC carrying 5′monophosphate (5′P) or triphosphate (5′PPP) (left panel). A sequencing gel (right panel) showing ^32^P-labeled products of the reactions from the left panel and mapping the RNase E (1-762)/RhlB cleavage sites in *ompD*. Lane 4 in both panels is a control sample loading of the *ompD*. The lanes labeled OH and T1 are ladders prepared by alkaline degradation and T1 nuclease digestion, respectively. (E) Schematic presentation of possible ways of RNase E cleavage of *ompD* mRNA in vitro. The arrows indicate the preferred cleavage positions in the presence and absence of MicC sRNA (purple). Sizes shown on the bottom panel indicate the lengths of the digestion products (U+113/114 cleavage site = 183/184 nts, A+98 = 168 nts, A+83 = 153 nts, A+72 = 142 nts products). The A+83 cleavage site is the preferred site in vivo (Pfeiffer et al., 2009). The schematic suggests cleavage preferences for the different pathways and not restricted activities. (See also Figures S2, S3, S4, and S5.)

MicC is a 109 nt sRNA which was found to regulate expression of the OmpD porin in *Salmonella* (Figures 2A and 2B). MicC basepairs with the CDS of the *ompD* transcript with the 12 nt ‘seed’ region located on the 5′ end of the sRNA (Figures 2B and 2C). In vivo, the targeted degradation of *ompD* involves RNase E, Hfq and MicC (Pfeiffer et al., 2009). An *ompD* cleavage product at position +83, relative to the AUG start codon (A is +1), is observed in a mutant strain expressing a truncated RNase E that encompasses the catalytic domain but lacks the scaffolding portion required to form the RNA degradosome (RNase E (1-701)). Like the artificial system presented in Figure 1, the A+83 site in *ompD* is a few nucleotides downstream of the region complementary to the MicC 5′ end (Figure 2B), suggesting that the sRNA might activate RNase E through a similar mechanism.

**Figure 3:**
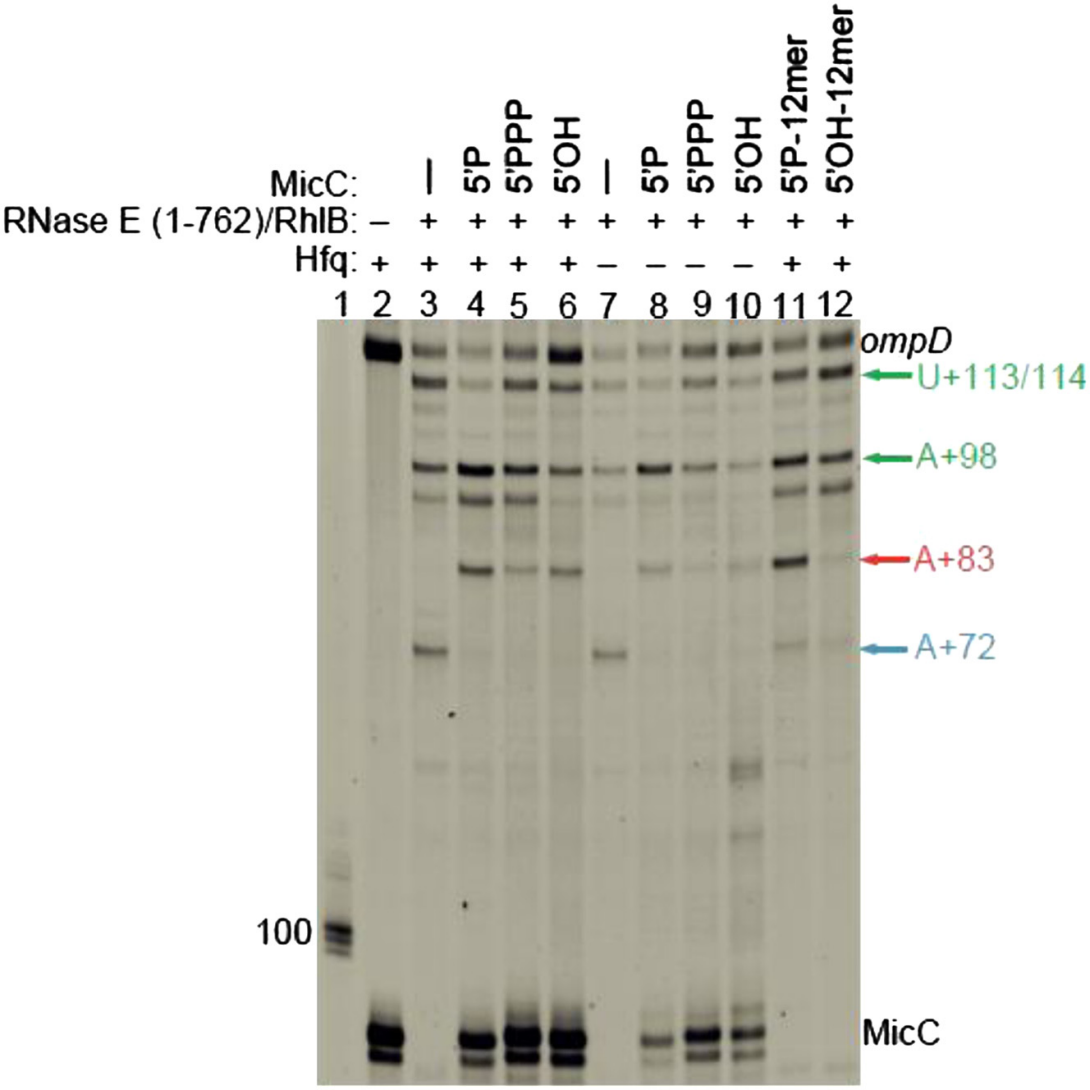
The Imperfectly Complementary “Seed Region” of MicC Is Sufficient to Induce RNase E Mediated Cleavage of *ompD*. Denaturing gel showing products of *ompD* degradation by RNase E (1-762)/RhlB with or without Hfq (lanes 3 and 7) and in the presence of WT MicC with different state of 5′ end (lanes 4-6 in presence of Hfq and 8-10 in absence of Hfq), as well as 12 nucleotides seed region of MicC (lanes 11-12). The RNA chaperone Hfq protects the sRNA and enhances cleavage at the A+83 site. Size markers are in lane 1, control reaction without enzyme is in lane 2. (See also Figures S6, S7, and S8.) The gel was stained with SYBR gold Nucleic Acid Gel Stain, which reveals all the RNA species. The lower band of MicC is a sRNA with 1 nt shorter polyU tail due to different transcription termination by T7 polymerase on polyT stretches.

We explored whether the *ompD* cleavage pattern could be recapitulated in vitro using purified *E. coli* Hfq and truncated RNase E in complex with helicase RhlB (hereafter, RNase E (1-762)/RhlB) (details in the Materials and Methods). This longer version of RNase E was found to have greater activity for *ompD* than the isolated catalytic domain, RNase E (1-529) (Figure S2, compare with Figures 2D and 3). RhlB is co-expressed with the RNase E 1-762 using a co-expression vector, and the helicase is required for stability of RNase E (Worrall et al., 2008). For these experiments, we used the full-length MicC RNA (Figure 2C) and a 187 nt section of the *ompD* transcript (nucleotides from −69 to +118 relative to AUG with an additional 5′ G) which encompasses the 5′ UTR and the first 39 codons of the CDS containing the MicC target site. Results presented in Figure 2D show the recombinant materials successfully mimic the cleavage pattern seen in vivo. In the presence of MicC that has a monophosphate group on the 5′ end (hereafter, 5′P-MicC), RNase E is able to generate the A+83 species under conditions of excess substrate over enzyme, while in contrast if the 5′ end of MicC carries a triphosphate group (hereafter, 5′PPP-MicC), the cleavage at the position A+83 is comparatively limited. These findings are consistent with the model that a 5′ monophosphate is favored by the enzyme. Corroborating the activating effects of a 5′-monophosphate, addition of recombinant pyrophosphohydrolase RppH to reaction mixtures containing 5′PPP-MicC boosts the formation of the +83 product from the *ompD* mRNA (results not shown). There are other cleavage sites observed by RNase E activity on the naked *ompD* RNA, such as the A+98 and U+113/114 sites, but cleavage products at these positions are not seen in vivo, therefore these sites must be inaccessible in vivo.

Based on the results shown in Figure 2D, we identify three main patterns for preferred RNase E cleavage in vitro of a 187 nt section of *ompD* mRNA in the presence of Hfq, shown in the general schematic of Figure 2E. The pathway when sRNA is absent is shown on the left; the middle depicts the preferences when the sRNA is present with a 5′P; and the right panel shows the preferences with the 5′PPP form of the sRNA. In all cases RNase E (1-762)/RhlB can cleave the *ompD* fragment at positions “upstream” (A+72) and “downstream” (U+113/114, A+98) of the A+83 site in A/U rich regions that are likely to be single-stranded. However, these cleavages are not activated as they occur for both mRNA alone and in the presence of sRNA.

When *ompD* is present in the reaction without MicC, the major RNase E (1-762)/RhlB cleavages are in position U+113/114 (results in 183/184 nt RNA fragment), A+98 (168 nt RNA fragment) and A+72 (142 nt product; Figure 2E left pathway). The presence of MicC results in protection of the A+72 site where sRNA binds and blocks the cleavage. However, when the sRNA carries a monophosphate on the 5′ end, the A+83 cleavage occurs more efficiently compared to the 5′PPP form of the sRNA.

The results of additional experiments to evaluate the RNA cleavage reaction are presented in the accompanying supplementary materials. Specifically, we confirm that our method of preparing 5′ monophosphate MicC is efficient (Figure S3). We also show that in the cleavage reaction, the generation of the A+83 product is not likely due to a contaminating nuclease, because the product is not seen in the presence of the catalytically inactive RNase E (1-762)/RhlB mutant D346R that was prepared with the same protocol as used for the native enzyme (Figure S4A); furthermore, it does not arise from an exoribonuclease activity, as no free rNDPs or rNMPs are produced by the cleavage reaction (Figure S4B). Hfq itself does not possess any nucleolytic activity, as no degradation products are observed after incubation of RNAs with Hfq (Figure S4A, lane without RNase E). Furthermore, we have also determined that the status of the mRNA 5′ end does not trigger the A+83 cleavage by RNase E (Figure S6).

Supporting the role of 5′ sensing by RNase E in the guiding effect, mutations in the 5′ sensing pocket (T170V and R169K) (Callaghan et al., 2005; Garrey et al., 2009) in the catalytic domain of RNase E (1-762)/RhlB resulted in loss of preference for the A+83 cleavage product for 5′P-MicC versus 5′PPP-MicC (Figure S5A). The mutants retained catalytic capacity per se, as they can accurately process 9S RNA into a 5S RNA precursor (Figure S5B). Taking the above results together, we conclude that the duplex formation and status of the 5′ end of the sRNA can guide RNase E to cleave preferred sites on a target mRNA.

### Hfq, a Monophosphorylated 5′ End, and a Seed Region of the sRNA Are Important for the RNase E Activation

We explored the effect of Hfq on the efficiency of RNase E (1-762)/RhlB to generate the guided cleavage product in vitro. Hfq, which is observed to have high affinity for *ompD* (Sittka et al., 2007), confers some protection of the naked transcript from cleavage, presumably by binding RNA in proximity of RNase E cleavage site (Figure 3, compare intensity of lanes 3 and 7). Hfq by itself is not sufficient to trigger RNase E to generate the A+83 product (lane 3). Its presence is also not strictly required for guiding RNase E, as 5′P-MicC is able to induce the A+83 cleavage in the absence of Hfq, albeit with lower efficiency (Figure 3, compare lanes 4 and 8). The phosphorylation state of *ompD* does not change the specificity of A+83 cleavage as 5′P-*ompD* is not capable of guiding RNase E to this site (Figure S6). Moreover, without Hfq present the intensity of the products of reaction changes, which indicates that the combination of both 5′P-MicC and Hfq are required for optimal RNase E activity in sRNA-guided mRNA cleavage.

To explore how MicC/*ompD* pairing might guide RNase E cleavage, we used the isolated 12 nt ‘seed’ region of MicC, which binds to the recognition site of *ompD* (Figure S7A; Pfeiffer et al., 2009). Like the full-length MicC, the 12-mer triggered cleavage of *ompD* by RNase E (1-762)/RhlB to form the specific A+83 fragment, and the reaction was more efficient when the 12-mer was monophosphorylated on the 5′ end; concordantly having a 5′-OH group on the seed region gave much weaker induction of the A+83 cleavage product compared with the 5′-P form (Figure 3, lanes 11 and 12, compare with lanes 4 and 6). The presence of Hfq slightly improves the efficiency of generating the A+83 product with the MicC 12-mer (Figure S7B). Similar weak inductive effects of a 5′-OH were seen for the full-length MicC (Figure 3, lane 6). Mutation of the cognate site in *ompD* abolishes the generation of the +83 site (Figure S8). These results show that seed region recognition is required for the guiding and activating effects, and that Hfq plays a supportive role in the process.

### 5′-End Status and mRNA Pairing Affect MicC Lifetime

**Figure 4:**
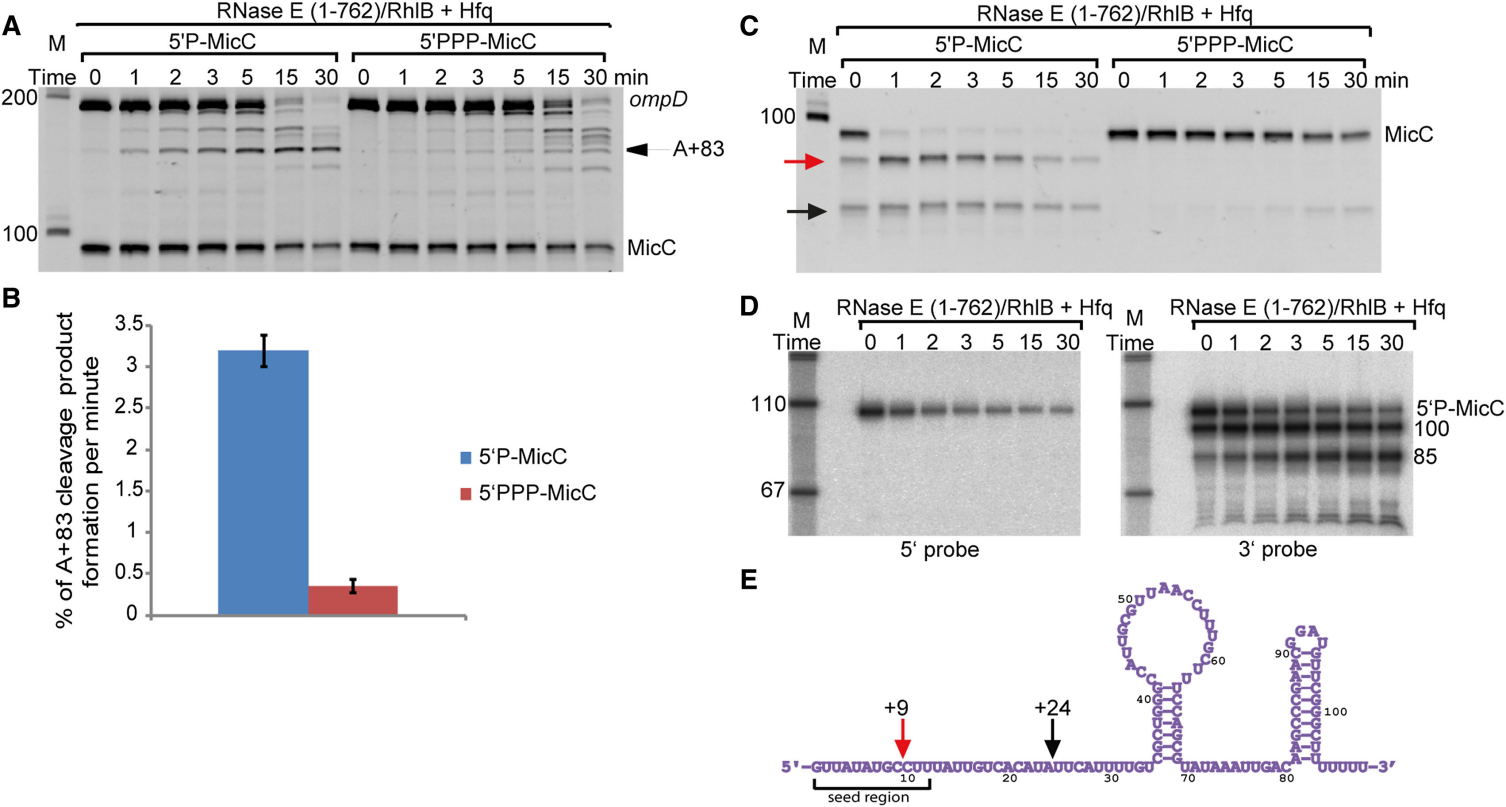
The Influence of MicC 5′ Phosphorylation State on Specific *ompD* Cleavage and MicC Stability (A) Time course series showing *ompD* degradation by RNase E (1-762)/RhlB in presence of Hfq and 5′P-MicC (left panel) or 5′PPP-MicC (right panel). (B) Graph representing efficiency of *ompD* A+83 cleavage by RNase E (1-762)/RhlB in presence of Hfq and 5′P or 5′PPP MicC, shown as the percent of the +83 cleavage (nM) per minute. Error bars were calculated for standard deviation for three independent experiments. (C) Time course series showing the influence of MicC 5′ phosphorylation state on its own degradation by RNase E (1-762)/RhlB in the presence of Hfq. The red arrow indicates the early cleavage product, and the black arrow indicates a second cleavage product. (D) Northern blot of the 5′P-MicC degradation as in left panel in (C), probed for 5′ end of MicC (left panel) and 3′ end of MicC (right panel). Size markers are in the left lanes (M), sizes of the cleavage products are indicated on the right. Minutes from the beginning of the reactions are indicated above the gels, 0 time point is a sample withdrawn about 5 s after enzyme addition. (See also Figure S9). (E) The cleavage sites corresponding to the red and black arrows in (C) were identified by 5′ RACE from four independent clones and are shown in the schematic of the MicC. The red arrow indicates the cleavage site within the seed region at position +9, and the black arrow indicates a second cleavage product at +24, just outside the seed region.

We compared the degradation rate and cleavage patterns of *ompD* and MicC by RNase E (1-762)/RhlB for the 5′P and 5′PPP forms of the sRNA in the presence of Hfq (Figures 4A and 4B). RNase E cleaves *ompD* fastest in the presence of 5′P-MicC, and while the mRNA fragment is also efficiently digested in the presence of 5′PPP-MicC, the preference for the A+83 site observed in vivo is significantly weaker. Expressed as relative efficiencies for the initial rates of formation of the A+83 product, 5′P-MicC has a 9 fold stronger effect compared with the 5′PPP-MicC (Figure 4B).

Both 5′P and 5′PPP forms of sRNA are slightly degraded by RNase E in the presence of *ompD*, but the enzyme shows a preference for 5′P-MicC (Figure S9, compare green and blue traces). However, the sRNA is cleaved slower than the mRNA (Figure S9). These data suggest that cleavage of the sRNA and mRNA may not be concomitant, but instead sequential. It is possible that MicC degradation is delayed until the digestion of *ompD* is initiated, when the sRNA is probably liberated. This hypothesis is also supported by the data presented in Figure S8, which shows rapid decay of the sRNA when it does not match the mRNA (*ompD* with the mutated seed pairing site).

The slower cleavage rates for the sRNA could in principle result from MicC not being a good substrate for RNase E; to examine this possibility, we tested degradation of MicC by RNase E (1-762)/RhlB in the presence of Hfq. MicC is protected from RNase E cleavage if it carries a 5′-triphosphate group, but becomes markedly liable to attack if the terminal group is a monophosphate (Figure 4C, compare left and right panels). The much greater susceptibility of the 5′P-MicC to degradation compared to 5′PPP-MicC is not due to weaker binding to Hfq; in fact, the former binds Hfq slightly better in fluorescent anisotropy binding assays, and both 5′P and 5′PPP forms when bound to Hfq have similar footprinting patterns by chemical and enzymatic probing (results not shown).

The 5′P form of the sRNA becomes strongly protected from RNase E attack when the mRNA target is present in the reaction, as can be seen from the decay profiles: there is complete degradation of the 5′P-MicC alone within 1 min, while in contrast only about 60% is lost in the presence of the target mRNA after 30 min (compare left panels of Figures 4A and 4C). The degradation products of 5′P-MicC by RNase E (1-762)/RhlB were analyzed by Northern blot. Samples from a reaction time course were divided and probed separately with the probe complementary to the 5′ end or 3′ end of MicC. The result indicates that first cleavage liberates the seed region of MicC (Figure 4D, compare left and right panels). The cleavage sites were mapped by 5′ RACE (Rapid Amplification of cDNA Ends) to positions +9 and +24 of the MicC sequence (Figure 4E). In contrast, when the 5′PPP is present, RNase E does not cleave at position +9, and only weak cleavage at the +24 site is observed, suggesting that the former cleavage site is preferred for 5′P-MicC. The removal of the seed region may serve as a means to rapidly inactivate the sRNA in the 5′P-state when its target is absent or when the suppression of target expression is no longer required. Hfq plays a very important role in this process as the +9 cleavage of 5′P-MicC occurs only in its presence, which indicate that Hfq not only recruits RNase E to the mRNA-sRNA duplex, but might expose sRNA cleavage sites when the sRNA is no longer needed and direct the sRNA on the degradation pathway.

### 5′P-MicC Can Be Detected In Vivo

**Figure 5:**
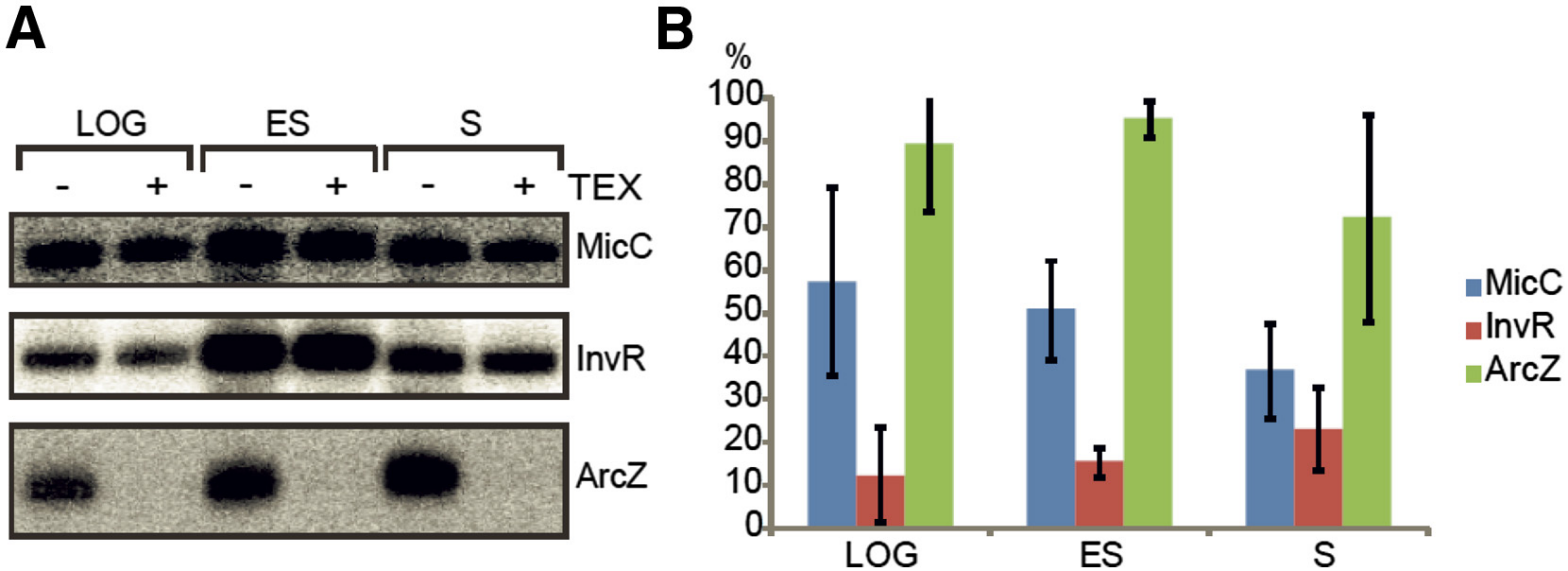
MicC in 5′P Form Can Be Detected In Vivo (A) Northern blot analysis of sRNAs from total RNA extracts of wild-type strain of *Salmonella*. The top panel shows the results for MicC at different cell growth densities (LOG, exponential phase, ES, early stationary phase; S, three hours into stationary phase). The same amounts of total RNA were either treated (+) with Terminator Exonuclease (TEX), which degrades RNA with a 5′ monophosphate, or untreated (−). Controls are shown for the sRNAs InvR, which is less TEX sensitive in logarithmic and exponential phases (middle panel), and ArcZ, which carries a 5′ monophosphate group and is sensitive to TEX treatment (bottom panel). (B) Bar chart representing the monophosphorylated fraction of sRNAs analyzed (MicC-blue, InvR-red, ArcZ-green), calculated from the percentage of integrated signal relative to the control not treated with TEX. Error bars were estimated from three independent experiments. (See also Figure S10.)

We tested if MicC is present in a 5′P form in vivo. The 5′ end status of MicC was evaluated using RNA extracted from cultures of *Salmonella*. MicC was detected by Northern blot analysis before and after treatment with Terminator Exonuclease (TEX), which will processively degrade RNA with a 5′ monophosphate group (Figure 5A). Controls with the sRNA InvR, which primarily has a 5′ triphosphate group (Pfeiffer et al., 2007), and the short form of ArcZ sRNA, which is processed from a precursor and has a 5′ monophosphate group (Argaman et al., 2001; Papenfort et al., 2009), confirm that the TEX enzyme is active and specific (Figure 5A).

The data indicate that there is a fraction of MicC in vivo which carries a 5′ monophosphate. This fraction is detectable in logarithmic (LOG), early stationary (ES), and prolonged stationary (S) phases of growth (between 36%–57%, Figure 5B). However, it is possible that a sRNA is maintained in the cell in 5′PPP form and the pyrophosphate removal is a consequence of stress conditions that induce sRNA action. We have tested a strain of *Salmonella* lacking the pyrophosphohydrolase RppH for MicC phosphorylation state, but the result obtained was very similar to those of the wild-type (data not shown). It may be that one of its numerous paralogues (Deana et al., 2008) is responsible for the pyrophosphate removal from MicC or replaces RppH function in its absence.

We also tested the 5′ end status of MicC associated with Hfq in vivo. The RNA co-immunoprecipitated with flag-tagged Hfq was treated with TEX, and the total MicC fraction bound to Hfq was compared with the TEX-treated sample on the Northern blot. The results shown in Figure S10 indicate that about 74% of the Hfq associated MicC is enriched in its 5′ monophosphorylated form. This would suggest that the activated form of sRNA can associate with Hfq in vivo, and supports the hypothesis that this ribonucleoprotein complex can guide RNase E specific cleavage of the mRNA target. There is a modest enrichment of the 5′P form of MicC on Hfq compared to its abundance in the total cellular MicC pool.

## Discussion

### Evidence for Specific, Accelerated mRNA Cleavage Programmed by sRNA

The predominant regulatory role of Hfq-dependent sRNAs is to suppress the expression of genetic information in a specific manner. Many of the *trans*-encoded targets are translationally repressed by their partner sRNAs, brought about by antisense-mediated sequestration of the ribosome binding site (RBS) or other sensitive regions of the targeted mRNA. The repression of translation often is concomitant with the destabilization of the target mRNA, and the latter is commonly regarded as a secondary consequence of the reduced protection conferred by ribosomes and the resulting vulnerability of the exposed transcript to ribonuclease attack.

**Figure 6:**
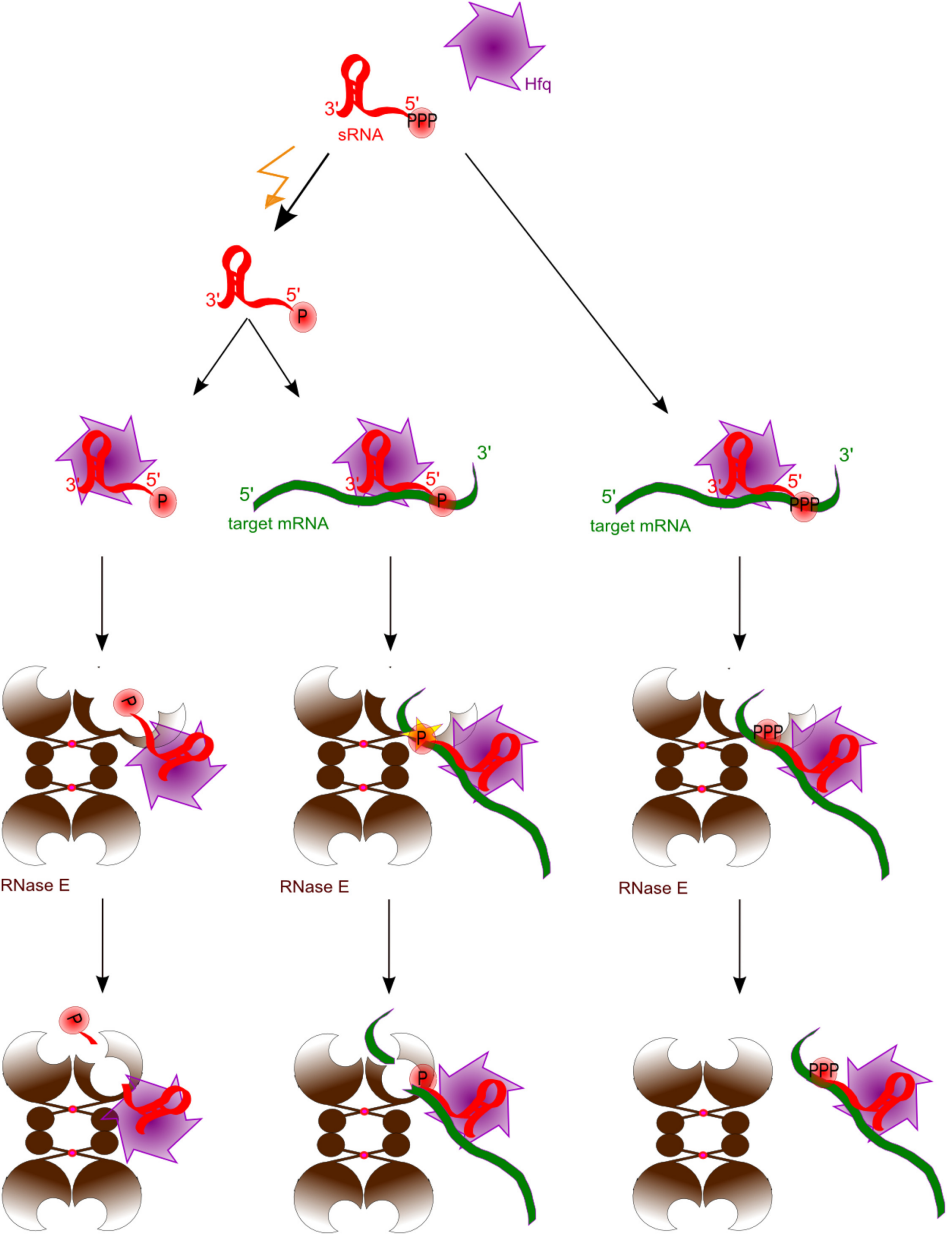
Cartoon Schematic of the Guide RNA Activation of RNase E and a Potential Proofreading Mechanism sRNA (red) is maintained in the cell in 5′PPP form. Such sRNA probably can associate with Hfq (purple) and basepair with a target mRNA (green), however 5′PPP group cannot actively stimulate RNase E (brown) (right panel). Under particular conditions (yellow lightening) the pyrophosphate from sRNA 5′ end is removed and such ‘activated’ sRNA (5′P marked with a star) efficiently guides RNase E cleavage of target mRNA (middle panel). 5′P form of sRNA would be rapidly degraded when there is no or no more target it can basepair with what would ensure ‘proofreading’ mechanism in sRNA mediated mRNA degradation.

The experimental data presented here reveal a pathway in which *trans-*acting sRNAs may potentially boost the decay rate of target mRNAs directly (Figure 6). It is possible that this pathway can operate in vivo without a primary requirement for translational repression. The inference for such a pathway is based on our findings that a sRNA can guide and activate RNase E to cleave a target mRNA six bases downstream of the seed recognition site. The activation occurs through 5′ end sensing by RNase E, which triggers a conformational switch in the enzyme and organizes the active site to accept a single-stranded substrate (Callaghan et al., 2005; Koslover et al., 2008). In the model proposed here the duplex formed between *trans*-acting RNA and the target RNA is an important aspect of RNase E recognition of substrate, and the 5′end of a *trans*-acting RNA may activate RNase E by interaction with the 5′ sensing pocket, whereas the cognate RNA with which it forms a duplex would be accommodated in the enzyme active site. Such activating effect is seen in both an artificial system (Figure 1) as well as the naturally occurring MicC:*ompD* system (Figures 2–5). The activation effect requires pairing of a region of complementarity between the sRNA and targeted transcript.

### The Degradosome and sRNA-Mediated Silencing

In vivo experiments in *Salmonella* suggest that the C-terminal portion of RNase E, which is the non-catalytic scaffold for the RNA degradosome, is important for sRNA mediated regulation. Deletion of this portion (residues 702 to 1061) weakens the repression of *ompD* by MicC (Pfeiffer et al., 2009). Other studies show that similar deletions decrease the degradation rate of the sRNA MicA 4-fold (Viegas et al., 2007), and diminish the effectiveness of RyhB-mediated silencing of *sodB* (Massé et al., 2003; Prévost et al., 2011).

The C-terminal portion of RNase E includes binding sites for helicase, enolase, PNPase and RNA (Górna et al., 2012; Kaberdin et al., 2000). Some of these may be important for mediating the repression effect, and one model proposes that these domains may help to recruit the Hfq:sRNA complex (Morita et al., 2004; Ikeda et al., 2011). The catalytic domain of RNase E is sufficient to observe the activating effect of a 5′-monophosphate group on the guide RNA MicC, but the efficiency is greater when RNase E includes a segment of the C-terminal portion that binds RNA and recruits the DEAD-box helicase RhlB (Figure S2). We observed that addition of ATP does not influence the apparent kinetics of formation of the A+83 product, suggesting that the ATP-dependent unwinding activity of the RhlB helicase is not required to generate the guiding effect of the sRNA, at least in vitro (data not shown). Binding data suggest that RNA can bridge between Hfq and the RNA-binding domains that are located in the C-terminal half of RNase E (Worrall et al., 2008). The interaction of these RNA-binding domains may help to present the seed region of sRNA, and also assist the delivery of the target to the catalytic domain of RNase E for cleavage. We envisage a mechanism for this action in which a sRNA activates the catalytic domain of RNase E, while other components of the degradosome assembly might interact with the target site to aid presentation to the active site.

### Temporal Order of Coupled Decay of Regulator and Target

sRNAs do not generally appear to be recycled, but instead are used only once and are degraded along with the target (Massé et al., 2003; Moll et al., 2003; Overgaard et al., 2009; Figueroa-Bossi et al., 2009). Our results suggest that cleavage of the sRNA and mRNA may not be concomitant, but perhaps instead synchronized, with sRNA cleavage following shortly once the mRNA has been cut (Figures 4 and S9). This would entail a mechanism for displacing the sRNA from the truncated mRNA. The results also indicate that sRNAs lifetime and activity can be modulated by the 5′ group (Figure 4C), and that the sRNA with a 5′P group may be comparatively more stable in the presence of the target. This suggests that sRNAs may be maintained in a protected form with a 5′-triphosphate group, and that there is a discard pathway if a partner is not met (Figure 6).

Most sRNAs studied to date accumulate as primary transcripts having a 5′PPP terminus, and processed sRNAs have not been so common. However, it is possible that the activated forms are not detected because they become rapidly degraded in this activated state. The activation could be either by processing a precursor form by RNase E, which generates an activating 5′P group, or by processing from a 5′-triphosphate to 5′-monophosphate by pyrophosphohydrolase, such as RppH, or one of its many paralogues in the Nudix superfamily (Deana et al., 2008).

### Fidelity and Response of sRNA

The proposed activating mechanism can account for many aspects of sRNA behavior that have been hitherto puzzling. Salient among these is the action of MicC at a position deep within the coding region, where it cannot possibly abrogate translation and therefore cannot regulate by impeding ribosome association (Pfeiffer et al., 2009). The model also explains how sRNAs trigger rapid instability of targets in vivo, e.g, RybB sRNA reduces the half-lives of stable *omp* mRNAs from ≥ 10 min to ∼1 min (Papenfort et al., 2006), which exceeds the decay rates caused by antibiotic-induced general block of mRNA translation (Sabina et al., 2003). Enhanced decay rates are manifested in the kinetics of target mRNA turnover mediated by other sRNAs of *E. coli* and *Salmonella* (Prévost et al., 2011; Guillier and Gottesman, 2008; Beisel and Storz, 2011; Durand and Storz, 2010; Johansen et al., 2008; Holmqvist et al., 2010; Vecerek et al., 2007). Our results may also account for how sRNAs acting far upstream of the translation initiation site may stimulate negative regulation of the mRNA target, as seen in the case of csgD mRNA regulation by the sRNAs OmrA, OmrB, and McaS (Jørgensen et al., 2012; Mika et al., 2012; Thomason et al., 2012; Holmqvist et al., 2010).

We observe that the activating effect of a 5′-monophosphate can be recapitulated with a short 12-mer RNA that corresponds to the seed region of MicC and so matches the cognate site in *ompD.* In general, the seed region of sRNAs is often surprisingly short, corresponding in some cases to an incomplete helical turn of duplex A-form RNA. Duplexes ≥ 7 bp formed between the complementarity segment and the recognition region can mediate sRNA interactions with a wide suite of targets (Papenfort et al., 2010; Balbontín et al., 2010; Guillier and Gottesman, 2008). Even in the cases of internal cleavage in the coding region, it is clear that the ‘seed’ region is not extensive (Pfeiffer et al., 2009; Fröhlich et al., 2012). The question naturally arises how the system achieves specificity as well as rapid response.

Taking our data together, we envisage a mechanism in which the 5′ end of an sRNA contributes to both the fidelity and speed of the responses that it mediates (Figure 6). For instance, if an activated sRNA, having a 5′P and associated with Hfq, does not match the cognate mRNA, or fails to find a target, then it can be rapidly degraded following RNase E cleavage, even in the presence of the chaperone Hfq (left branch, Figure 6). However, in the presence of the target mRNA, the sRNA bearing a 5′P guides cleavage of the transcript and in turn is protected from attack until after the target has been cleaved (central branch, Figure 6). The combination of these processes is expected to increase the fidelity and kinetics of sRNA-mediated response, and it bears some analogy to the energy-dependent kinetic proofreading mechanism that increases the fidelity of translation. In that proofreading mechanism, GTP hydrolysis is used to discriminate a mismatched codon: anticodon pair in the ribosome to ensure faithful protein synthesis (Wohlgemuth et al., 2011). In the case of sRNA, it is the turnover of non-coding RNAs that are activated but not paired that ensures increased specificity of sRNA action. Our findings thus suggest a role of 5′ end status on sRNA to control both flux and activity to achieve high fidelity and rapid responses to environmental signals.

## Experimental Procedures

### Preparation of *E. coli* RNase E(1-529) Catalytic Domain and the RNase E (1-762)/RhlB Complex

The RNase E catalytic domain (residues 1-529) was overexpressed and purified as described by Callaghan et al., 2005. The RNase E 1-762 F575E/helicase complex was expressed from a pRSF_rne762rhlB vector (Worrall et al., 2008). IPTG induced cells were harvested and lysed with an EmulsiFlex-05 cell disruptor (Avestin). The lysate was clarified by centrifugation and the soluble fraction was loaded onto Ni-NTA HisTrap column (GE-Healthcare), washed extensively with HisTrap buffer A (50 mM Tris-HCl, pH 7.8, 1 M NaCl, 5 mM imidazole, 5 mM MgSO_4_, 5 mM β-mercaptoethanol, 5% (v/v) glycerol, 1 tablet/500 ml EDTA-free protease inhibitor cocktail (Roche)) and eluted with a gradient of HisTrap buffer B (HisTrap buffer A supplemented with 0.5 M imidazole). Fractions enriched with RNase E/RhlB complex were dialysed against Heparin buffer A (50 mM Na-phosphate, pH 7.9, 250 mM NaCl, 10 mM DTT, 5% (v/v) glycerol) and loaded on 5 ml HiTrap Heparin column (GE Healthcare), and eluted with a gradient of Heparin buffer B (Heparin buffer A supplemented with 2 M NaCl). Enriched fractions were concentrated and fractionated from a S200 column (GE Healthcare) in buffer composed of 50 mM Tris-HCl, pH 7.9, 0.5 M NaCl, 50 mM KCl, 1 mM MgCl_2_, 5 mM DTT, 5% (v/v) glycerol, 1 tablet/1 L EDTA-free protease inhibitor cocktail (Roche).

RNase E mutants (R169K, T170V, D303R, D346R, D303RD346R) for both the catalytic domain (residues 1-529) and truncated degradosome (residues 1-762) were prepared using the QuikChange Site Directed Mutagenesis protocol (Stratagene). All purified protein samples were > 95% pure judging from SDS gel electrophoresis.

### Preparation of *E. coli* Hfq

Hfq was purified as described by Worrall et al. (2008) and Vassilieva et al. (2003). The lysate from centrifugation steps was kept at 22°C to minimize precipitation.

### Preparation of RNA

RNA was obtained by in vitro transcription (IVT) according to standard protocol (MicC, *ompD*) or chemically synthesized (12-mers MicC, 5′FAM-27-mer, guide 13mers). Plasmids carrying *Salmonella micC* or *ompD* genes (Pfeiffer et al., 2009) were used as a template in PCR reactions to prepare templates for IVT. PCR reactions were performed with primers complementary to amplified gene, simultaneously adding promoter sequence recognized by T7 RNA polymerase used in IVT, as well as in case of *ompD* G on 5′ end to enhance IVT efficiency. For synthesis of monophosphorylated RNA, five-fold excess of GMP over GTP was used, which resulted in capping the RNA products with high efficiency (see Figure S3). Products of IVT reactions were purified on 8% polyacrylamide gel with 7M urea. Bands containing RNA were visualized by UV–shadowing and excised. RNA was recovered from gel slices by overnight electroelution with an EluTrap System (Whatman).

Radiolabelled RNA was prepared by IVT in presence of α^32^P-UTP using Mega Script Kit (Ambion). Sample was purified by gel electrophoresis, eluted for 12 hr from gel slices and precipitated with ethanol in presence of NaOAc.

### RNA Assays

Degradation assays were performed in buffer containing 25 mM Tris pH 7.5, 50 mM NaCl, 50 mM KCl, 10 mM MgCl_2_, 1 mM DTT and 0.5 U/μl RNaseOUT (RNase E assays) or 10 mM potassium phosphate buffer pH 7.5, 1 mM MgCl_2_, 20 mM Tris pH 7.5, 1 mM DTT and 1 U/μl RNaseOUT (PNPase assays). Degradation assays were carried out in 37°C. 0.2 μM each RNA (time course reactions), or 0.4 μM each RNA (30 minutes reactions), or 0.3 μM RNA oligo and 0.05 μM enzyme were used per 10 μl reaction. Before addition of enzyme, RNA was heated for 2 min in 50°C, slowly cooled to room temperature and incubated with Hfq for 10 min at 37°C (1:1:1 molar ratio MicC:*ompD*:Hfq). Time course reactions were stopped after 0, 1, 2, 3, 5, 15 and 30 min, other reactions after 30 min by incubation with Proteinase K in 50°C for 15 – 30 min. Reactions with FAM labeled RNA where performed with concentrations 5 μM 5′FAM-27-mer, 5.7 μM guide RNA and 200 nM RNase E (1-529), and stopped after 0, 2, 5, 10, 15 and 30 min by incubation with Proteinase K. 0 time point was taken from the reaction mixture after the enzyme was added and responds to 10-15 s. RNA loading dye (Fermentas) was added and after denaturation in 95°C for 3 min whole samples were loaded on polyacrylamide gels with 7M urea. Gels were stained in SYBR Gold solution (Invitrogen) and RNA was visualized with GeneSnap and quantified with GeneTools (Gel documentation and analysis system from Syngene).

Reactions visible in the right panel of Figure 2D were prepared as described above but after incubation with Proteinase K RNA was phenol-chloroform extracted, precipitated, dephosphorylated with CIP (NEB) and labeled with γ^32^P-ATP in the presence of PNK (Fermentas).

### RNA isolation and Northern Blot Analysis

RNA was extracted and analyzed as described by Pfeiffer et al., 2009. Co-immunoprecipitation was carried out from bacteria cultures at OD_600_ = 2 according to protocol in Pfeiffer et al., 2007. Treatment with terminal exonuclease (TEX, Epicentre) was done as described by Sharma et al. (2010). All Northern blots were repeated in triplicate.

### In Vitro Footprinting

In vitro footprinting was performed with 5 mM Pb^+2^ as in Pfeiffer et al., 2009.

### 5′ RACE

5′ RACE (Rapid Amplification of cDNA Ends) experiments were carried out as described by Urban and Vogel (2007) with omission of the TAP treatment step.

## Supplemental Information

Document S1. Ten Figures and One Table
