# Supplementary material for: The Seed Region of a Small RNA Drives the Controlled Destruction of the Target mRNA by the Endoribonuclease RNase E
Source: Mol Cell. 2012 Sep 28;47(6):943–53. doi: 10.1016/j.molcel.2012.07.015 (PMC3469820; doi:10.1016/j.molcel.2012.07.015)
Supplement: Document S1. Ten Figures and One Table [file mmc1.pdf]

Molecular Cell, Volume *47*

Supplemental Information

The Seed Region of a Small RNA Drives the Controlled Destruction of the Target mRNA by the Endoribonuclease RNase E

Katarzyna J. Bandyra, Nelly Said, Verena Pfeiffer, Maria W. Górna, Jörg Vogel, and Ben F. Luisi

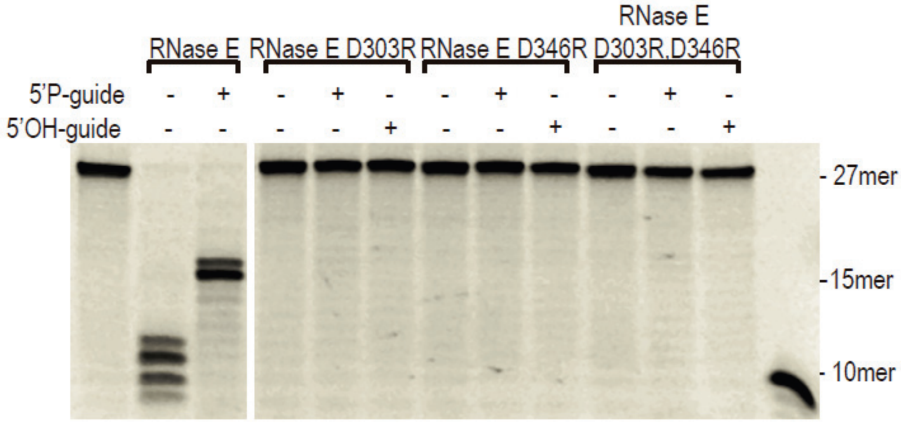

Figure S1, Related to Figure 1. Cleavage of 27-mer with a 5' fluorescent label (FAM) in the presence or absence of unlabelled guide 13-mer RNA by RNase E (1-529) WT and inactive catalytic mutants: D303R, D346R and double D303R,D346R. The guide 13-mer RNA used in the experiment has 2'O-methyl ribose modifications throughout to protect against RNase E attack, showing that the directed internal cleavage of the 27-mer does not require cleavage within the 13-mer guide. The lane on the far right is a fluorescent-labelled 10-mer size marker.

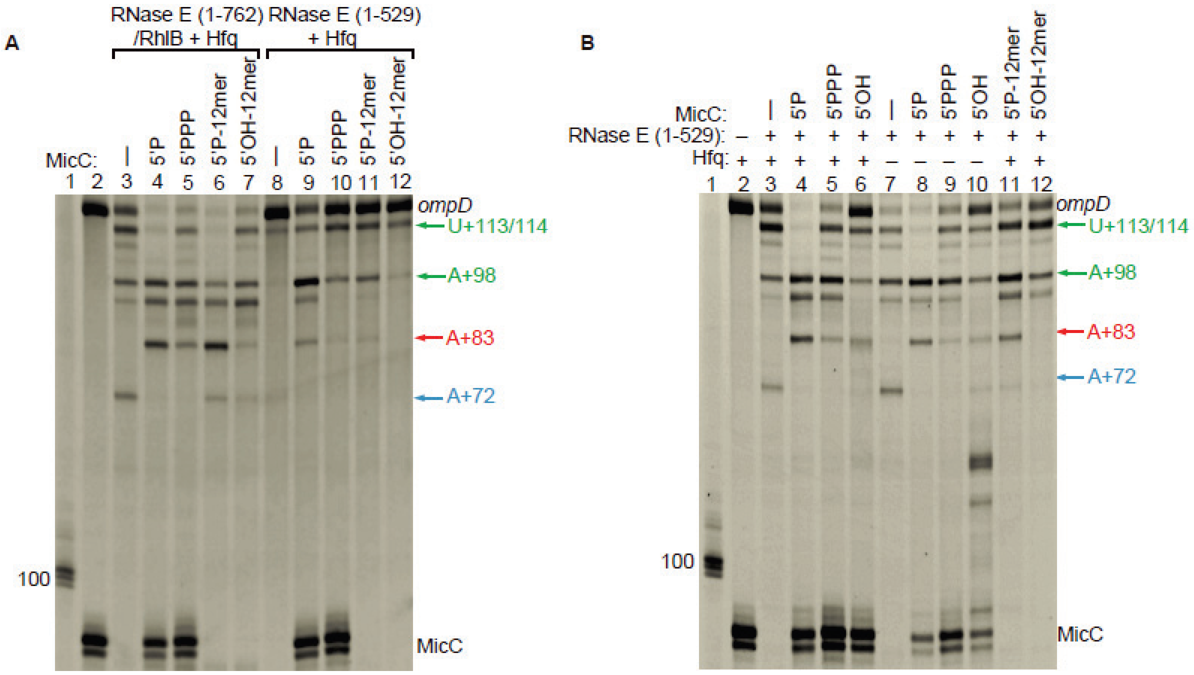

Figure S2, Related to Figures 2D and 3. Comparison of the activity of RNase E (1-529) and RNase E (1-762)/RhlB against *ompD* in the presence and absence of MicC sRNA and/or Hfq. (A) The comparison of the rates of catalysis of the two RNase E constructs at 50 nM and four-fold excess of substrate over enzyme. (B) A duplicate of the experiment shown in Figure 3 from the main text, except that RNase E catalytic domain (residues 1-529) was used rather than RNase E (1-762)/RhlB. The concentration of RNase E (1-529) is 150 nM, i.e., 3 times greater than the 50 nM concentration used for RNase E (1-756)/RhlB, since the former has lower activity.

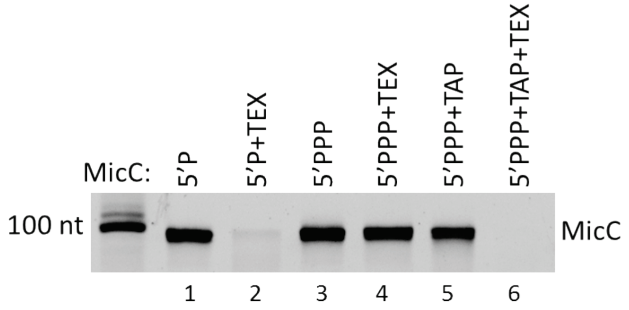

Figure S3, Related to Figure 2. Efficiency of *in vitro* transcription (IVT) to generate 5’ monophosphate MicC. 5’P-MicC and 5’PPP-MicC RNAs prepared by IVT were treated with Terminator Exonuclease (TEX; lanes 2 and 4). As a control, 5’PPP-MicC was transformed into 5’P form with Tobacco Acid Pyrophosphatase (TAP), followed by subsequent treatment by TEX (lanes 5 and 6).

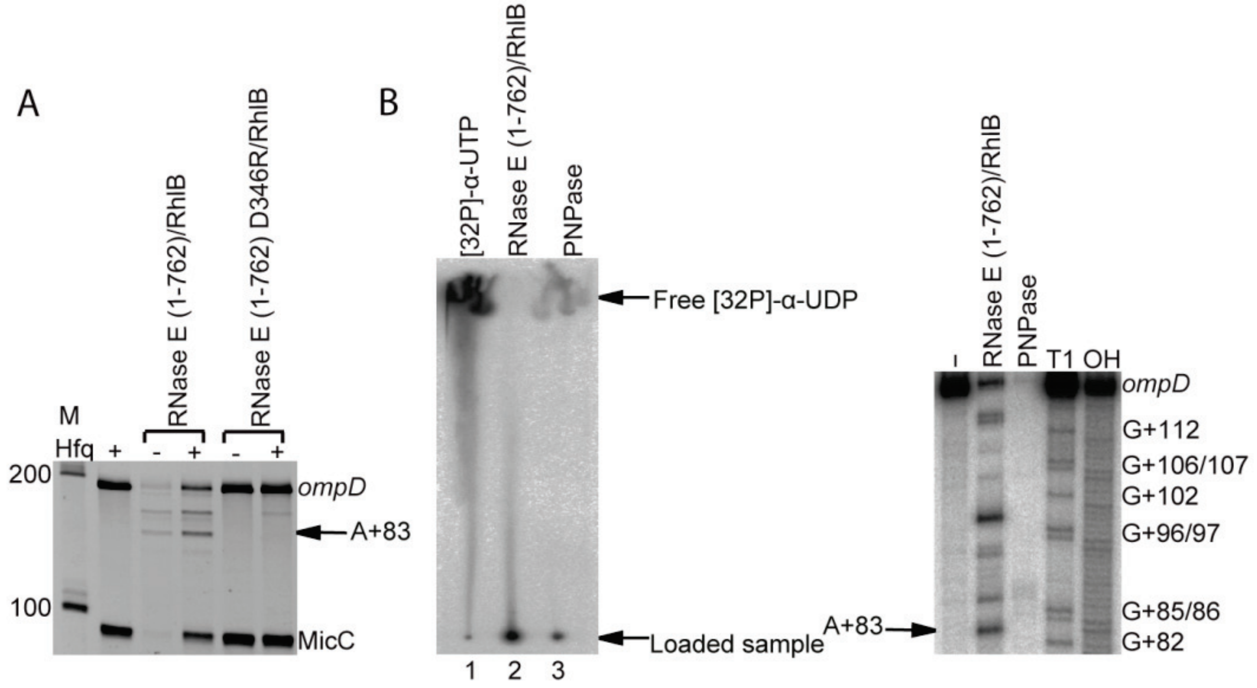

Figure S4, Related to Figure 2. The detected *ompD* cleavage events are not due to a contaminating nuclease. A) 30 minutes incubation of 5'P-MicC and 5'PPP-*ompD* with RNase E (1-762)/RhlB wild type or catalytic site mutant D346R.The specific cleavage of *ompD* at the position A+83 is absent when RNase E is inactivated by mutation and cannot be restored by Hfq. B) (Left panel) Thin layer chromatography results for reactions of 5'P-MicC and 5'PPP-*ompD* with RNase E (1-762) or purified recombinant PNPase. While rNDPs could be detected for PNPase (lane 3), neither rNDPs or rNMPs were detected in RNase E reaction, which confirms lack of exonuclease activity for the latter. (Right panel) As a control, the same reactions as on left panel were analysed on 6% sequencing gel. The A+83 product is visible only in reaction where RNase E is present.

Reactions for TLC were loaded on PEI Cellulose TLC plates (Macherey-Nagel), dried and developed in 1.5M potassium phosphate buffer pH 7.5 for about 3 hours.

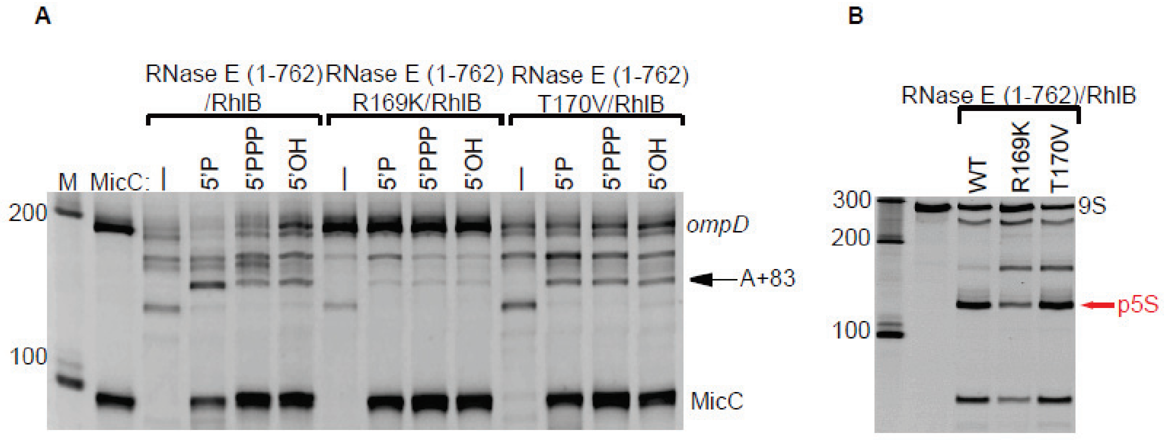

Figure S5, Related to Figure 2. The 5' sensing pocket of RNase E is required for discrimination of sRNA and guided mRNA decay. (A) RNase E (1-762)/RhlB wt (left panel) is sensitive to the 5' end status of MicC and cleaves *ompD* efficiently in position +83 in the presence of 5'P-MicC. RNase E (1-762)R169K/RhlB and RNase E (1-762)T170V/RhlB are insensitive to the sRNA phosphorylation state and cannot process *ompD* efficiently. (B) Control reactions for RNase E (1-762)/RhlB wt and mutants R169K and T170V showing that all enzymes are capable of processing 9S RNA to p5S RNA, which is the precursor for mature 5S.

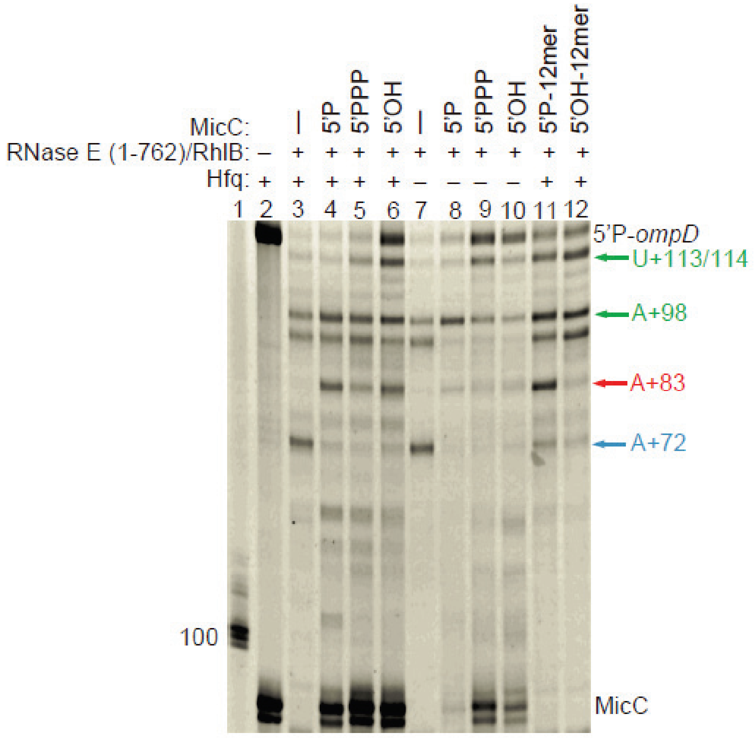

Figure S6, Related to Figure 3. 5' end of *ompD* is not important for A+83 cleavage. The cleavage of 5'P-*ompD* alone and in presence of different MicC variants by RNase E (1-762)/RhlB; lanes are in the same order as presented in Figure 3. The 5' end of mRNA influences only the rate of the cleavages but does not guide RNase E.

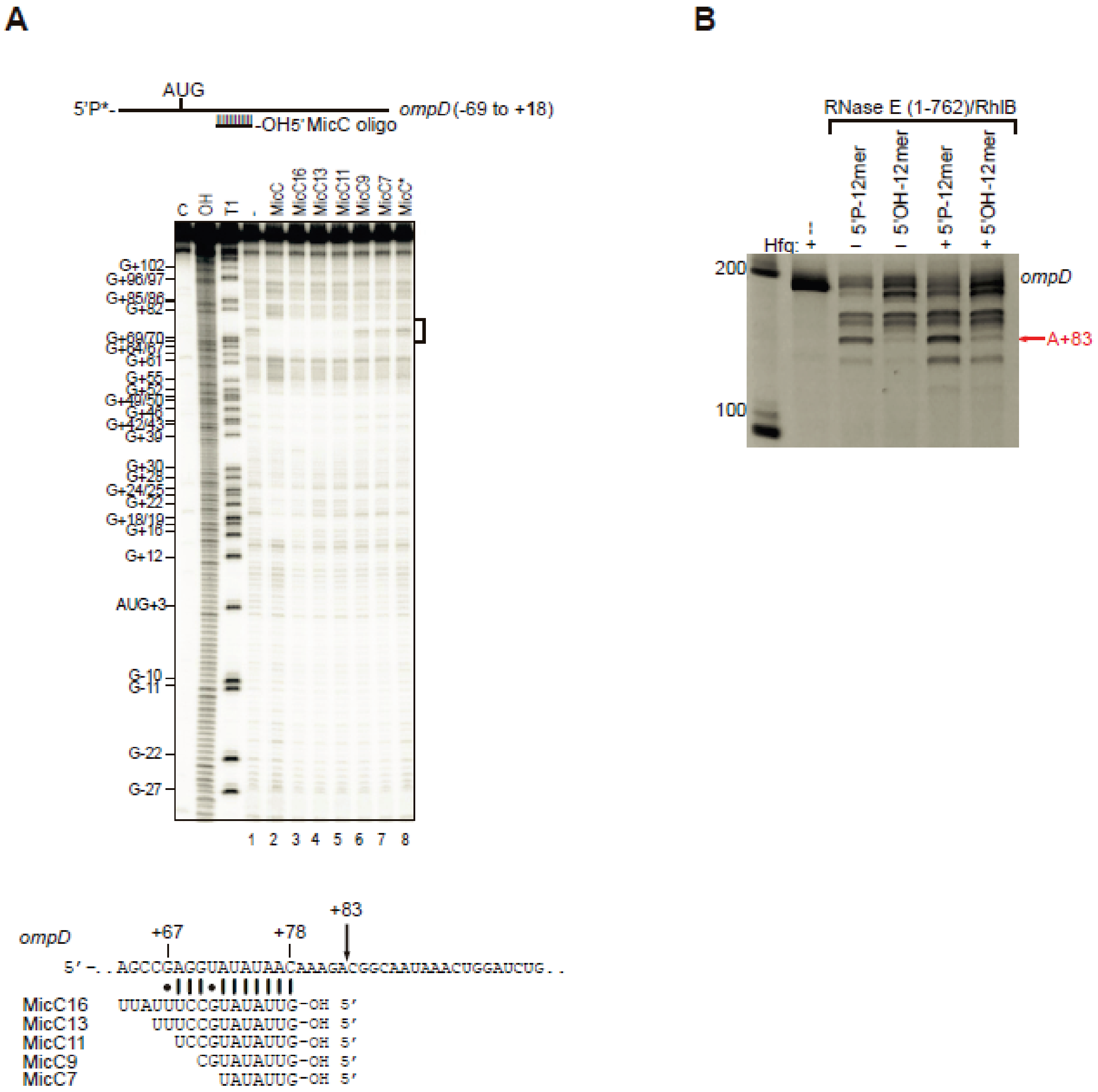

Figure S7, Related to Figure 3. The seed region of MicC basepairs with *ompD* and triggers RNase E cleavage. A) Footprinting experiment showing different MicC seed lengths sufficient for formation of MicC-*ompD* duplex. The bracket on the right encompasses the region of ompD that is protected. B) Products of the RNase E (1-762)/RhlB cleavage of *ompD* in presence of 12 nts long MicC seed region without and with Hfq. Lane labeled M has markers and C is the control of *ompD*. The RNA chaperone has modest influence on the reaction efficiency when only the seed region of MicC is used.

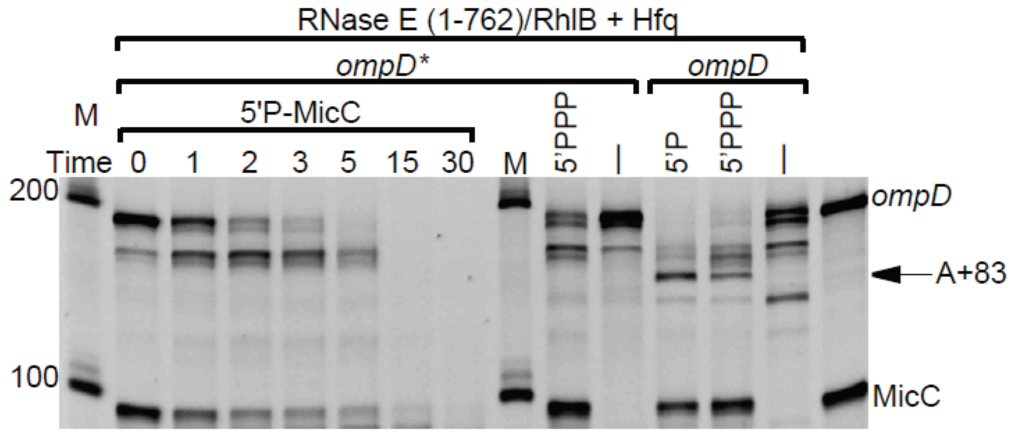

Figure S8, Related to Figure 3. Mutation of the cognate site in *ompD* abolishes the generation of the +83 site and causes rapid degradation of unpaired sRNA. RNase E (1-762)/RhlB cleavage timecourse of *ompD** and MicC in the presence of Hfq (left panel; time in minutes) shows that the +83 cleavage cannot be induced without duplex formation between the sRNA seed and the mRNA recognition site. Without the basepairing between sRNA and mRNA, the sRNA is rapidly destroyed in contrast to gaining stability when the seed region matches mRNA (see Figure 4). The right panel presents the controls with 5'PPP-MicC and wild type *ompD*. *ompD** is *ompD* with mutation G+70C (Pfeiffer et al., 2009). The last lane is a control reaction without RNase E.

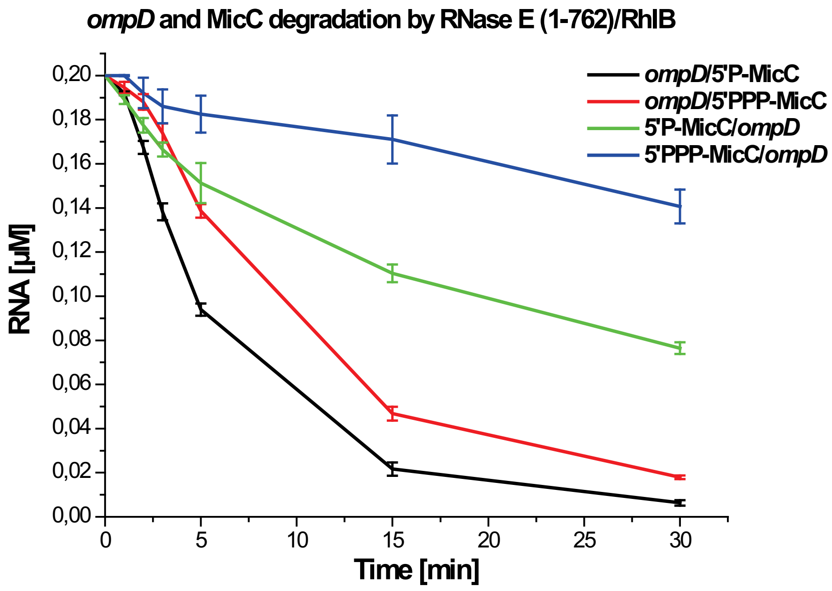

Figure S9, Related to Figure 4. *ompD* and MicC degradation by RNase E (1-762)/RhlB. The decay of *ompD* and MicC was analysed in 3 repetitions of timecourse reactions for degradation in the presence of 5’P-MicC or 5’PPP-MicC. Black trace-*ompD* degradation in presence of 5’P-MicC, red trace-*ompD* degradation in presence of 5’PPP-MicC, green trace-5’P-MicC degradation in presence of *ompD*, blue trace-5’PPP-MicC degradation in presence of *ompD*.

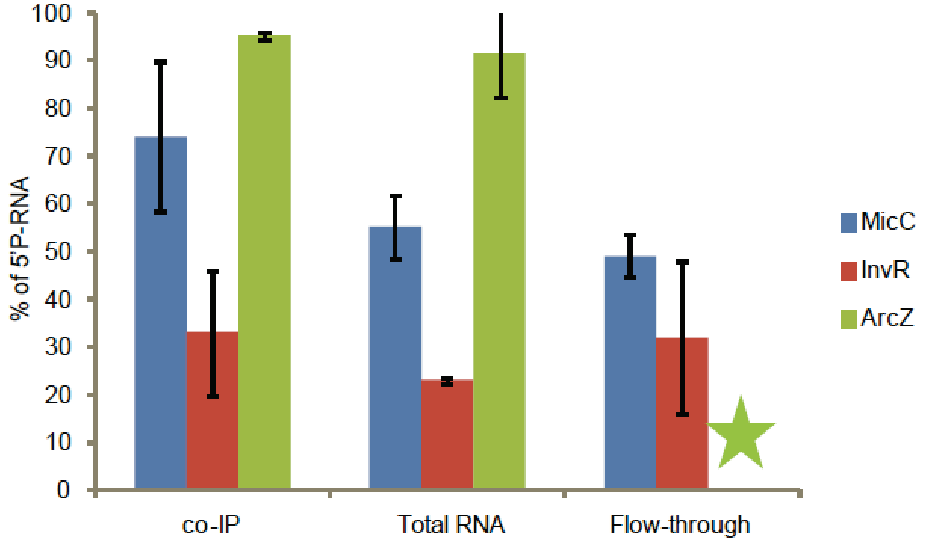

Figure S10, Related to Figure 5. Quantitated Northern blot analyses of the fraction of 5’P forms of MicC, ArcZ and InvR that co-immunoprecipitate with Hfq in a wild type strain of *Salmonella*. The signals were compared for samples that were treated with TEX with untreated controls. The flow-through is the sample that eluted through the affinity matrix. These data suggest that Hfq associated MicC can be in the 5’P state. This form may be modestly enriched over the unbound form. The green asterisk for the ArcZ sample in the co-IP flow-through signifies that it was undetectable.

Table S1. Quantification of the +83 digest product

	ompD alone		*ompD*+5'PMicC		*ompD*+5'PPP-MicC		*ompD*+5'OH-MicC		*ompD*+5'P-12mer MicC	*ompD*+5'OH-12mer MicC
	+Hfq	-Hfq	+Hfq	-Hfq	+Hfq	-Hfq	+Hfq	-Hfq	+Hfq	+Hfq
ompD cleaved (nmole)	153.4 ±22.9	174.1 ±20.9	167.7 ±12.0	160.2 ±28.8	119.2 ±40.6	141.5 ±24.8	91.9 ±24.0	128.4 ±27.9	182.6 ±11.2	160.4 ±3.9
+83 product (nmole)	-	-	29.3 ±3.4	12.7 ±6.4	8.9 ±3.6	3.5 ±0.5	14.6 ±5.1	6.7 ±3.0	32.8 ±13.3	3.7 ±0.7

The products were measured at a fixed time point (30 minutes). The cleaved *ompD* was estimated by subtracting the integrated signal corresponding to the full length from the value of the control sample incubated without enzyme (200 nmole). The band intensities were integrated from images recorded with a Syngene gel imagining system using GeneTools software.
